# How deep is deep enough? The importance of the detection limits of the CML minimal residual disease gDNA assay

**DOI:** 10.1038/s41420-026-03221-9

**Published:** 2026-06-26

**Authors:** Giovanni Micheloni, Vittoria Moretti, Annalisa Frattini, Francesco Acquati, Giorgio Binelli, Clara Belotti, Chiara Francesca Longo, Lucy Costantino, Martina Milani, Tamara Intermesoli, Rolland A. Reinbold, Giuseppe Montalbano, Orietta Spinelli, Roberto Valli, Giovanni Porta

**Affiliations:** 1 Dipartimento di Medicina e Chirurgia, Università dell’Insubria, 21100 Varese, Italy; 2 Istituto di Ricerca Genetica e Biomedica (IRGB), Consiglio Nazionale delle Ricerche (CNR), Milan, Italy; 3 Dipartimento di Biotecnologie e Scienze della Vita, Università dell’Insubria, 21100 Varese, Italy; 4 Hematology Unit, ASST Papa Giovanni XXIII Hospital, Bergamo, Italy; 5 FROM Research Foundation, ASST Papa Giovanni XXIII, Bergamo, Italy; 6 CDI Centro Diagnostico Italiano, Laboratory of medical genetics, via Saint Bon 20, Milano, Italy; 7 Institute of Biomedical Technologies, National Research Council of Italy, 20054 Segrate, Italy; 8 Present Address: Centro di Medicina Genomica, Università dell’Insubria, 21100 Varese, Italy

**Keywords:** Chronic myeloid leukaemia, Cancer genomics

To the editor,

The introduction of imatinib (Gleevec/Glivec or STI-571), the first Tyrosine Kinase Inhibitor (TKI), revolutionized the management of Chronic Myeloid Leukemia (CML), dramatically improving patient quality of life and life expectancy, which now approaches that of the general population [1].

Although TKIs only allow an “operational cure”, primarily due to their inability to eradicate quiescent BCR::ABL1^+^ leukemic cells (LCs) [2], the high number of patients reaching deep molecular response (DMR) has shifted the clinical management from how to treat them to how to identify who can safely interrupt therapy, i.e., how to determine treatment free remission (TFR) [3].

In this *scenario*, minimal residual disease (MRD) evaluation by RT-qPCR, performed on mRNA extracted from peripheral blood [4], becomes essential to assess patient status and to direct therapeutical decisions on discontinuation.

Despite very high sensitivity, RNA-based techniques require fully validated tests and continuous controls because different factors can alter proper mRNA quantification [4]. In contrast, DNA-based approaches—for example, gDNA breakpoint-specific qPCRs—are generally more reliable [5].

Several discontinuation trials have been performed to identify the conditions that allow therapy interruption and durable TFR [6], ending up in a quite defined *scenario*: around 50–60% of the total number of CML patients can become candidates for interrupting therapy, and around half of these will experience relapses, restricting TFR to 25–30% of all CML patients [7].

These findings have compelled researchers to explore the clinical and biological factors influencing TFR outcomes [1, 3, 6].

The Centre of Genomic Medicine at the University of Insubria (Varese, Italy) suggested analyzing DNA breakpoint sequences for MRD monitoring back in late 2000s [8, 9], through a qPCR patient-specific assay designed on characterized breakpoint region sequence [8–10].

This assay is patient-specific and directly counts LC, regardless of their transcriptional status [9].

Previous studies comparing qRT–PCR and gDNA qPCR on CML patients showed a higher sensitivity and specificity of the DNA-based approach to monitor MRD in long-term follow-ups [11–13].

In this study, we compared MRD obtained by RT-qPCR and gDNA qPCR on follow-up from a cohort of patients monitored for 15 years on average. We repropose the statistical analysis described by Glauche and coworkers [14], on data obtained via gDNA at different sensitivities, to verify how the sensitivity can affect MRD evaluation (Supplementary Information SI1 - Materials and Methods).

In addition, we pushed the analysis of MRD by gDNA to a MR^6^ level, deeper than what is currently obtainable with mRNA.

We have previously described the patients enrolled in this study [13], and Tables 1 and 2 in Supplementary Information SI2 summarize patients’ clinical data and therapeutic history.

We analyzed a total of 287 follow-ups using both qRT-PCR and gDNA qPCR. DNA was detected in 90.6% (260/287) of samples (gDNA sensitivity 10^-6^), while mRNA in 45.6% (131/287) (mRNA sensitivity at 10^-4^). Among follow-ups with undetectable mRNA (156/287), 86.5% of samples were gDNA positive (135/156).

MRD evaluations for representative patients are depicted in Fig. 1, graphs for each patient are available in Supplementary Information SI3 and LC counts are available in Supplementary Information SI4.

**Fig. 1:**
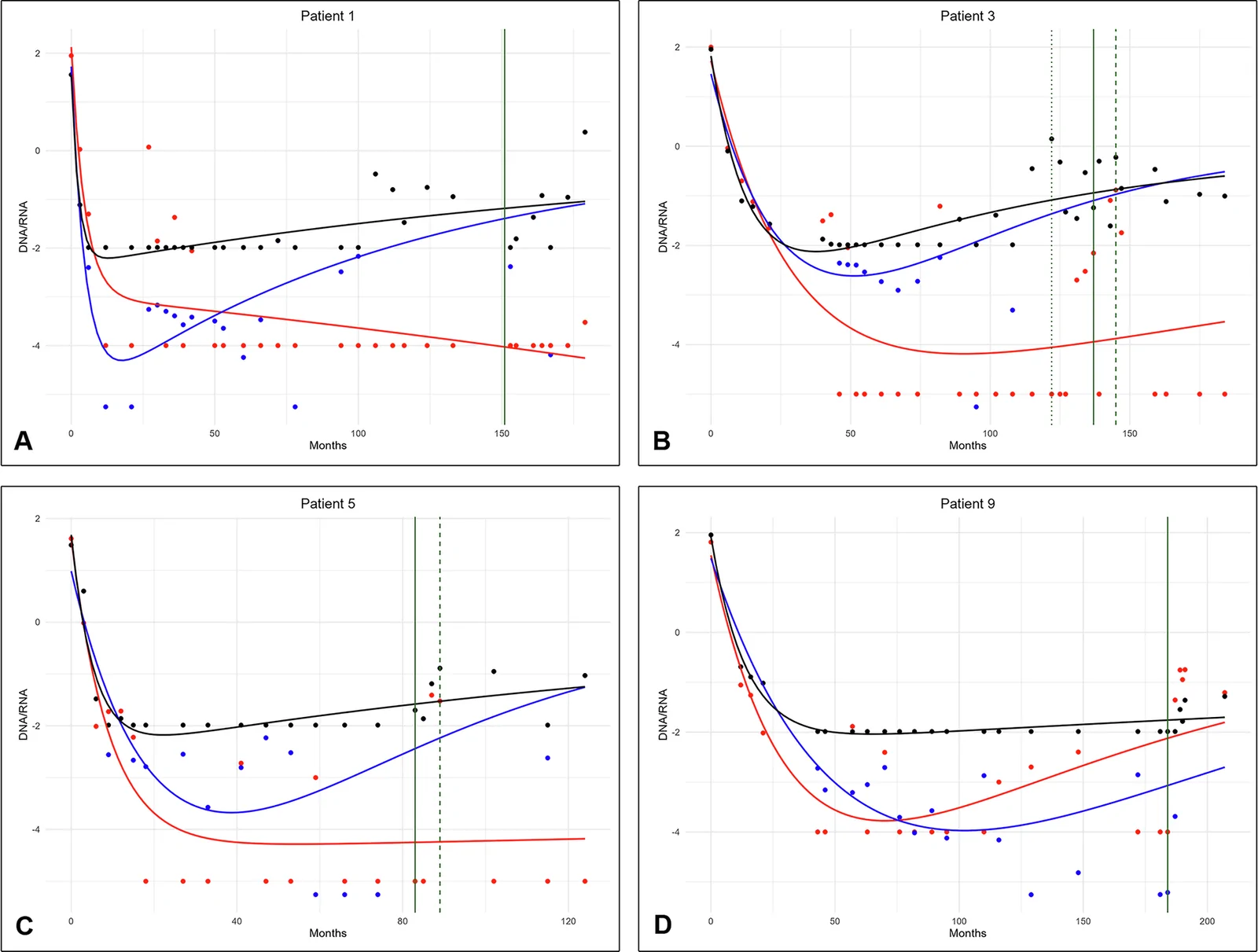
Monitoring of MRD of representative patients. **A** Follow-ups of patient 1. **B** Follow-ups of patient 3. **C** Follow-ups of patient 5. **D** Follow-ups of patient 9. Patients 1 and 9 interrupted TKI without needing to resume it. Patients 3 and 5 interrupted therapy and started back after losing DMR. Dots represent follow-ups, interpolated by bi-exponential curves; the blue curve is gDNA detected at a sensitivity of 10^-6^, the red curve is mRNA, the black curve is gDNA detected at a sensitivity of 10^-4^. The green solid vertical line indicates the month at which patients interrupted therapy; the green dashed vertical line indicates when patients started back TKI assumption; the green dotted vertical line in patient 3 graph indicates reduction of imatinib dosage from 400 mg/day to 200 mg/day. X-axis: months from diagnosis. Y-axis: log10 of the percentage of leukemic cells or *BCR::ABL1* (%IS).

We hypothesize that a good response to imatinib treatment led to eradication of transcriptionally active cells in most patients, while LSCs survived in bone marrow niche, due to its microenvironment and resulting in insensitivity toward therapy [2], and then enter the bloodstream.

Among the eight patients interrupting therapy, five presented less than 100 cells at the time of discontinuation (pt. 1, 4, 7, 8 and 9), two between 100 and 1 000 cells (pt. 3 and 5) and one presented more than 1 000 cells (pt. 10). (Fig. 1 and Supplementary Information SI4).

Based on the cell counts in the follow-ups before discontinuation, patients can be divided in two groups: the first group (pt. 7, pt. 8, pt. 9, and pt. 10) displayed low levels of leukemic cell counts in the year before discontinuation (below 1 000 per million), while the second group (pt. 1, pt. 3, pt. 4 and pt. 5) exhibited higher levels of leukemic cells (above 1 000 per million).

The patients from the first group maintained an LC number below thousands of cells per million and MR^5^ levels at mRNA analysis till the last follow-up, while those from the second group show an increase in both mRNA and gDNA levels a few months after discontinuation, forcing them to restart imatinib (pt. 3, 4 and 5), or reaching a peak of 24 000 cells per million (pt. 1) (Fig. 1 and Supplementary Information SI4).

Patient 6, surprisingly, maintains high levels of both mRNA and gDNA throughout all 16 years of treatment without signs of disease progression or therapy failure, maybe due to *BCR::ABL1*expression in a different lineage [15].

Statistical analysis on MRD data confirmed that mRNA and gDNA curves are better described by a bi-exponential curve, as previously described [14], as stated by lower Akaike information criterion (AIC) (Table 3 in Supplementary Information SI2).

Comparison between mRNA and gDNA curves was statistically significant in all patients except pt. 9, suggesting that the two methods describe two distinct events.

The comparisons between the curves obtained at two gDNA sensitivities (10^-4^ and 10^-6^) were significant in six out of ten patients (patients 1, 2, 5, 8, 9 and 10; Table 4 in Supplementary Information SI2). Interestingly, these different curves were not clearly associated with TFR outcomes. Moreover, we observed a deeper response toward TKI in patients with significantly different curves resulting in a reduction of ≥10-4 in LC count (100 LCs / 1 000 000 cells).

These data further strengthen the idea that MRD evaluated with gDNA provides a deeper and broader image of the leukemic burden, which is essential to properly evaluate when and how to interrupt therapy.

No direct correlation between LC numbers at discontinuation and TFR was observable, considering that patients relapsing presented low- and mid-cell numbers, while pt. 10 harboring the highest number of cells at discontinuation did not relapse after 57 months.

We also observed that patients with less than 1 000 cells/1 million in the year before discontinuation maintained TFR, while those presenting higher LCs failed discontinuation.

We are aware that to determine whether gDNA-based LC trend could be an effective predictor of TFR, it is mandatory to broaden this analysis to more patients, in order to collect more robust statistical evidence. The need for patients with long-term follow-ups, currently in therapy or discontinuation trials, with DNA collection at diagnosis and at each follow-up, limited the analysis to patients enrolled from the beginning of the study.

A protocol to enroll a higher number of patients is ongoing by our laboratory.

In conclusion, although the identification of patients that can safely interrupt TKI therapy remains complex, we believe that it is important to perform DNA analysis alongside mRNA to gain a complete picture of the patient’s status. To optimize the gDNA approach costs, we suggest extracting and storing DNA from peripheral blood at diagnosis. In patients who reach DMR during the treatment, the breakpoint sequence should be characterized, and the patient specific gDNA assay should be designed. This is a “patient-specific MRD genomic marker” that should be used in parallel with the standard mRNA approach for a therapy discontinuation decision.

## Supplementary information

Suppmenetary Information

## Data Availability

The data presented in this study are available on reasonable request from the corresponding author at roberto.valli@uninsubria.it.
