# Supplementary material for: How deep is deep enough? The importance of the detection limits of the CML minimal residual disease gDNA assay
Source: Cell Death Discov. 2026 Jun 26;12:363. doi: 10.1038/s41420-026-03221-9 (PMC13562504; doi:10.1038/s41420-026-03221-9)
Supplement: Supplementary file 1 [file 41420_2026_3221_MOESM1_ESM.zip › Supplementary Information SI1 - Materials and Methods.docx]

**MATERIALS AND METHODS**

**Patient Enrollment**

The patients enrolled in this project were receiving care at ASST Papa Giovanni XXIII (Bergamo, Italy). Human samples were obtained and analyzed in accordance with the declaration of Helsinki, after written consent. All protocols were approved by the Ethics Committee of Papa Giovanni XXIII Hospital (Bergamo, Italy).

Patients received first-line therapy with imatinib mesylate 400 mg/day, except for patient 6 that received 800 mg/day.

**Breakpoint Characterization**

The breakpoints of these patients were characterized in 2008 by nested PCR [44]. Briefly, genomic DNA was fragmented, ligated to adaptors, amplified through nested PCR with forward primers specific for *BCR* exon 13 and reverse primers binding to the adapter, and finally sequenced by Sanger.

**Follow-up Sampling**

Follow-up samples were collected at Bergamo Hospital. Follow-up timing was determined based on baseline risk, transcript level, and transcript level fluctuations. Blood samples were processed to eliminate red blood cells and erythroid precursors, as previously described [45], and divided into aliquots for subsequent analysis. The MRD was evaluated using either qRT-PCR and gDNA qPCR for each follow-up.

**qRT-PCR**

qRT-PCR analyses were carried out in the Hematology laboratory of the Hematology Division of ASST Papa Giovanni XXIII (Bergamo), following European LeukemiaNet recommendations.

Total RNA was extracted from follow-up aliquots using the RNA blood kit (Qiagen, Hilden, Germany). The mRNA analysis was performed using the M-bcr FusionQuant kit (Ipsogen, Marseille, France), approved for clinical diagnosis, according to manufacturer’s protocol. The kit has a sensitivity of at least 10^-4^ dilution of *BCR::ABL1*-positive RNA in a negative RNA sample.

MR was assessed, according to the BCR::ABL1^IS^, as the ratio of *BCR::ABL1* transcripts to *ABL1* transcripts.

**gDNA-qPCR**

DNA was extracted from follow-up aliquots using the FlexiGene DNA Kit (Qiagen, Hilden, Germany), as described in [63,64], and stored at -20°C.

The BP of each patient was previously characterized and patient-specific gDNA assays were developed. A control assay, amplifying a region in the *BCR* where breakpoints do not occur, was designed, and tested on patient’s onset DNA and on DNA from a healthy donor.

Patient-specific assays were re-tested on onset DNAs to confirm assay sensitivity and specificity.

The percentage of leukemic cells in the follow-up samples was calculated through a modification of the formula described by Mattarucchi [45]: in this study we used a control assay that always amplifies two copies of *BCR* per cell, either in normal or leukemic cells. For this reason, the formula to calculate the percentage of leukemic cells has been modified to: LC = 100 · (2/2ΔCt).

gDNA qPCR reactions were set up in 96-well plates: the control assay was performed in triplicate, while *BCR::ABL1* amplifying reactions were set up to analyze a total of ~6µg of DNA (divided into 300ng for each well with a total amount of 20 well for each reaction), corresponding to ~1x106 cells, to reach a MR^6^ level of 1 LC/1 000 000.

**Statistical Analysis**

Following the work by Glauche and colleagues [48], we described a patient’s treatment response by modelling the nonlinear relationship between the time under treatment (t) and the level of DNA (RNA) by the logarithm of a bi-exponential function—i.e.: DNA (RNA)(t) = log10 (A−αt + B−βt), with parameters A, B, α, and β—which is common in decay or growth analysis with two different phases. The parameters A and B can be interpreted as the intercepts of the two approximating line segments, while α and β represents the slopes of these lines. The significance of each curve was tested twice: first, by checking whether the parameters A, α, B, and β were significantly different from zero; second, by checking whether the double exponential model fit the data better than a simpler model (such as a single exponential), using a Likelihood Ratio Test. Fitting the curves was performed in nls.lm in the standard nls framework of R (package minpack.lm). Comparisons between different curves were also carried out using an LRT using nls.lm.
