# Supplementary material for: How deep is deep enough? The importance of the detection limits of the CML minimal residual disease gDNA assay
Source: Cell Death Discov. 2026 Jun 26;12:363. doi: 10.1038/s41420-026-03221-9 (PMC13562504; doi:10.1038/s41420-026-03221-9)
Supplement: Supplementary file 1 [file 41420_2026_3221_MOESM1_ESM.zip › Supplementary Information SI2 - Tables.docx]

| **Table 1: Clinical data of patients enrolled in the present study** | | | | | | |
| --- | --- | --- | --- | --- | --- | --- |
| **Patient** | **Sex** | **Age at diagnosis**  **(yr)** | **Diagnosis**  **(DD/MM/YY)** | **Translocation** | **Therapy Start**  **(DD/MM/YY)** | **Therapy** |
| Pt 1 | M | 46 | 21/02/2006 | t(9;22)(q34;q11)  p210 e13a2 | 07/03/2006 | IM 400 mg/day |
| Pt 2 | M | 41 | 02/11/2010 | t(X;9;22)(p11;q34;q11)  p210 e13a2 | 05/11/2010 | IM 400 mg/day |
| Pt 3 | M | 47 | 20/05/2005 | t(9;22)(q34;q11)  p210 e13a2 | 25/05/2005 | IM 400 mg/day |
| Pt 4 | M | 37 | 30/03/2005 | t(9;22)(q34;q11)  p210 e13a2 | 12/04/2005 | IM 400 mg/day |
| Pt 5 | M | 67 | 12/07/2010 | t(9;22)(q34;q11)  p210 e14a2 | 16/07/2010 | IM 400 mg/day |
| Pt 6 | F | 58 | 03/02/2005 | t(9;22)(q34;q11)  p210 e13a2 | 14/02/2005 | IM 800 mg/day |
| Pt 7 | F | 60 | 16/12/2010 | t(9;22)(q34;q11)  p210 e14a2 | 21/12/2010 | IM 400 mg/day |
| Pt 8 | F | 50 | 02/12/2004 | t(9;22)(q34;q11)  p210 e13a2 | 21/12/2004 | IM 400 mg/day |
| Pt 9 | M | 38 | 08/10/2004 | t(9;22)(q34;q11)  p210 e13a2 | 12/10/2004 | IM 400 mg/day |
| Pt 10 | M | 46 | 30/05/2005 | t(9;22;16)(q34;q11;q24)  p210 e13a2 | 08/06/2005 | IM 400 mg/day |

**Table 1:** clinical data of patients enrolled into the study. M: male; F: female; yr: year; IM: imatinib.

| **Table 2: therapy administration** | | | | |
| --- | --- | --- | --- | --- |
| **Patient ID** | **Therapy dosage** | **Dosage variation/month** | **Therapy discontinuation** | **Therapy reassumption** |
| 1 | 400 mg/day | - | month 151 | - |
| 2 | 400 mg/day | - | - | - |
| 3 | 400 mg/day | 200 mg/day  month 122 | month 137 | 200 mg/day  month 145 |
| 4 | 400 mg/day | - | month 192 | month 196 |
| 5 | 400 mg/day | - | month 78 | month 89 |
| 6 | 800 mg/day | 400 mg/day / month 65 | - | - |
| 7 | 400 mg/day | - | month 88 | - |
| 8 | 400 mg/day | 600 mg/day /month 12;  400 mg/day /month 73 | month 146 | - |
| 9 | 400 mg/day | - | month 172 | - |
| 10 | 400 mg/day | 600 mg/day /month 6;  400 mg/day /month 44 | month 139 | - |

**Table 2:** imatinib therapy. The table summarizes imatinib dosages assumed by each patient, highlighting variation in dose and therapy discontinuation timepoints.

| **Table 3 : Likelihood ratio test** | | |
| --- | --- | --- |
| **Patient** | **gDNA vs gDNA§** | **AIC** |
| 1 | 8.622e-05 *** | 160 / 99 |
| 2 | 7.687e-07 *** | 113 / 61 |
| 3 | 0.253 n.s. | 145 / 94 |
| 4 | 0.236 n.s. | 125 / 67 |
| 5 | 0.0394 * | 113 / 78 |
| 6 | 1 n.s. | 96 / 32 |
| 7 | 0.4924 | 112 / 91 |
| 8 | 3.004e-09 *** | 194 / 105 |
| 9 | 5.221e-06 *** | 136 / 81 |
| 10 | 0.864 n.s. | 98 / 56 |

**Table 3**. *p*-values of Likelihood-Ratio Tests used to compare the bi-exponential curves obtained from gDNA (detected at sensitivity level of 10^-6^, this work) and gDNA§ (detected at sensitivity level of 10^-4^). AIC = Akaike Information Criterion for mono-exponential and bi-exponential curves (this order) for gDNA. * = *p* < 0.05; *** = *p* < 10^-3^, n.s. = non-significant.

| **Table 4: significance of curves vs. TFR outcome.** | | |
| --- | --- | --- |
|  | **Statistically Significant** | **Not statistically Significant** |
| **TFR: no relapse** | Pat. 1, 8, 9, 10 | Pat. 7 |
| **TFR: relapse** | Pat. 5 | Pat. 3, 4 |
| **No therapy discontinuation** | Pat. 2 | Pat. 6 |

**Table 4:** Patients that relapsed or non-relapsed after TFR in relationship to the significance of the gDNA curves comparison at 10^-4^ or 10^-6^
