# Supplementary material for: How deep is deep enough? The importance of the detection limits of the CML minimal residual disease gDNA assay
Source: Cell Death Discov. 2026 Jun 26;12:363. doi: 10.1038/s41420-026-03221-9 (PMC13562504; doi:10.1038/s41420-026-03221-9)
Supplement: Supplementary file 1 [file 41420_2026_3221_MOESM1_ESM.zip › Supplementary Information SI3 - MRD evaluation.pdf]

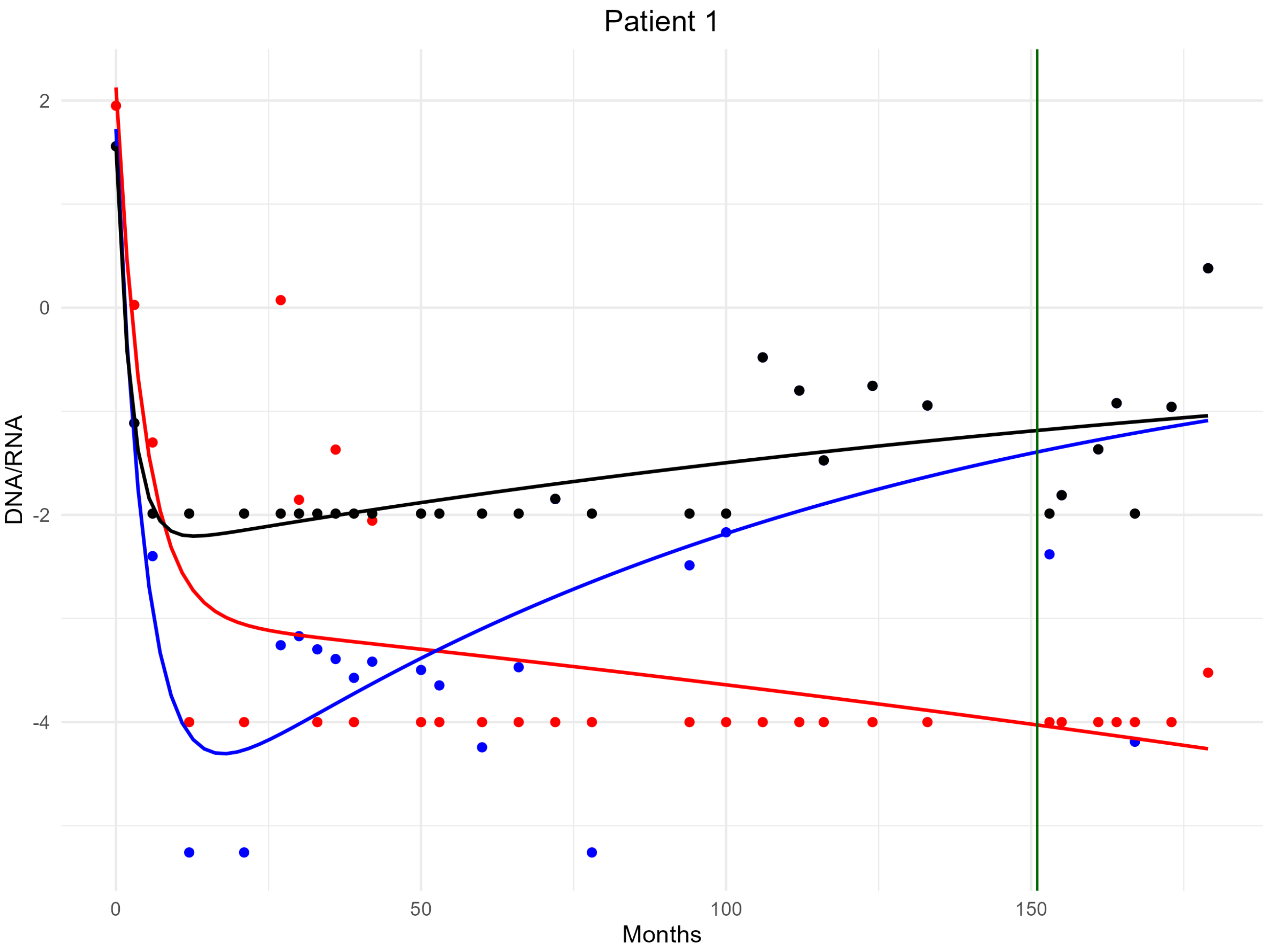

SI4: MRD monitoring of patient 1. Dots represent follow-ups, interpolated by bi-exponential curves; the blue curve is gDNA detected at a sensitivity of 10^{-6}, the red curve is mRNA, the black curve is gDNA detected at a sensitivity of 10^{-4}.

The solid green vertical line indicates the month at which patient interrupted therapy (151).

X-axis: months from diagnosis. Y-axis: log10 of the percentage of leukemic cells or *BCR::ABL1* (%IS).

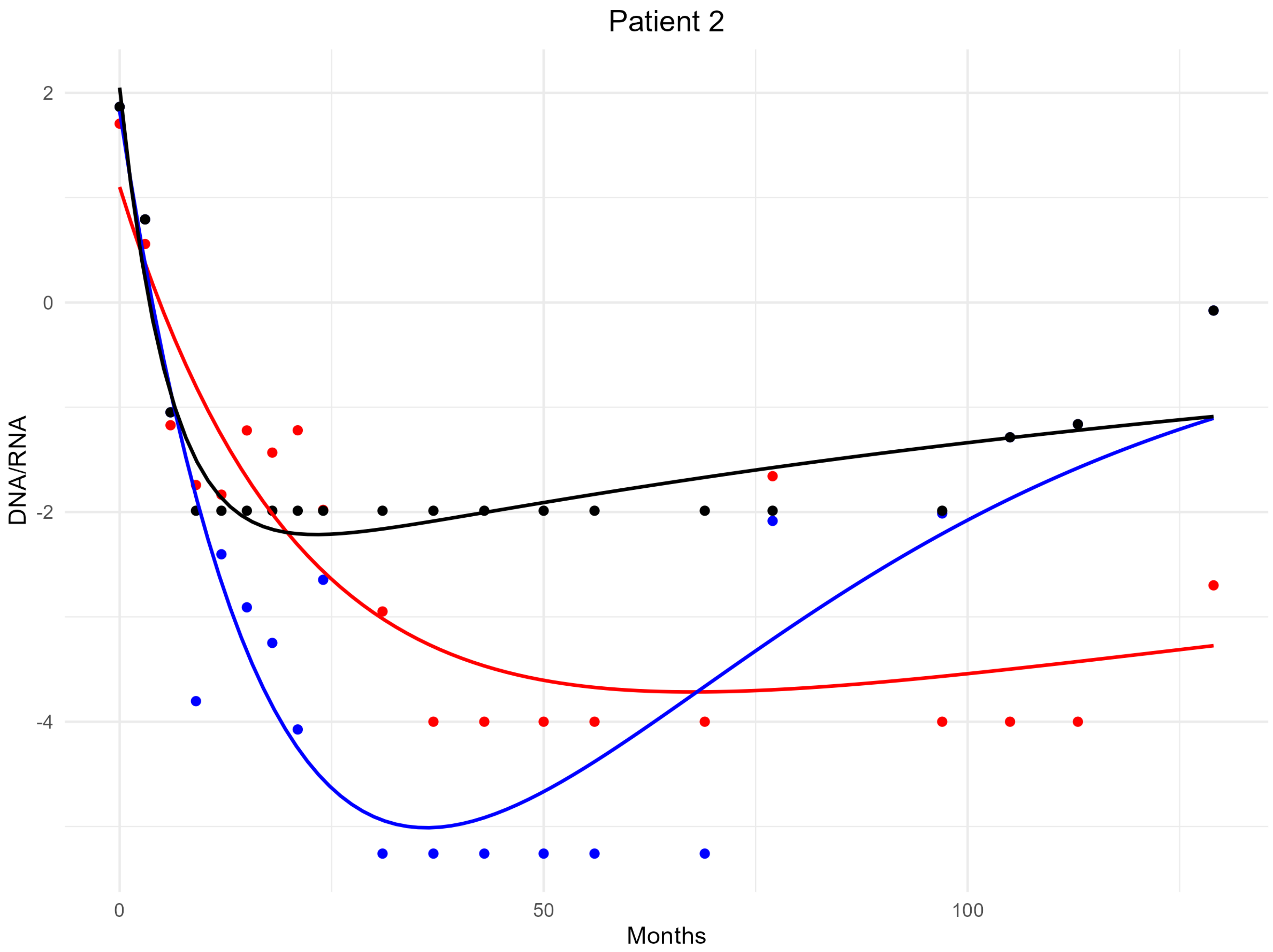

SI4: MRD monitoring of patient 2.

Dots represent follow-ups, interpolated by bi-exponential curves; the blue curve is gDNA detected at a sensitivity of 10^{-6}, the red curve is mRNA, the black curve is gDNA detected at a sensitivity of 10^{-4}.

X-axis: months from diagnosis. Y-axis: log10 of the percentage of leukemic cells or *BCR::ABL1* (%IS).

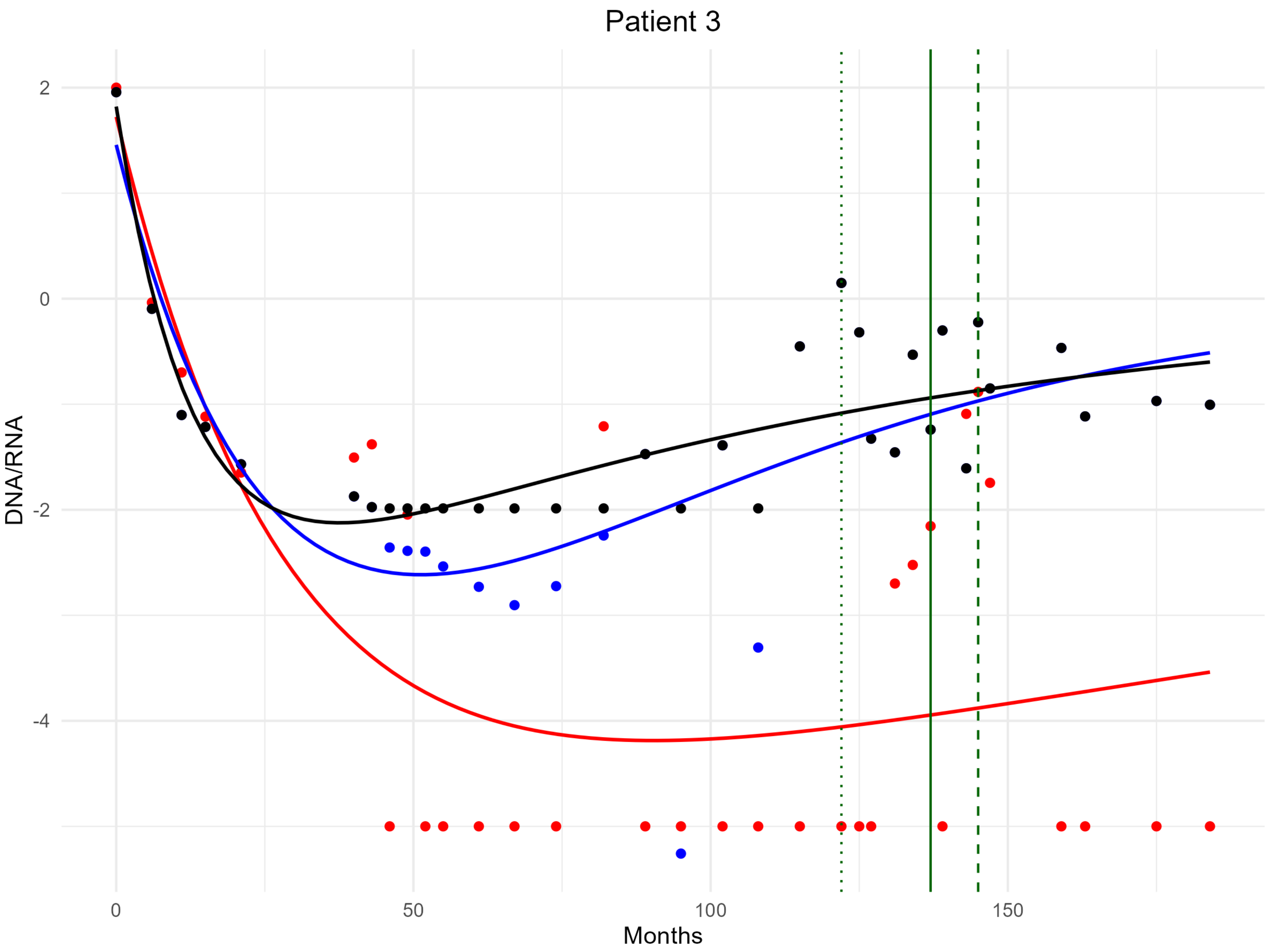

SI4: MRD monitoring of patient 3.

Dots represent follow-ups, interpolated by bi-exponential curves; the blue curve is gDNA detected at a sensitivity of 10^{-6}, the red curve is mRNA, the black curve is gDNA detected at a sensitivity of 10^{-4}.

The green solid vertical line indicates the month at which patient interrupted therapy (137); the green dashed vertical line indicates when patient started back TKI assumption (145); the green dotted vertical line indicates reduction of imatinib dosage from 400 mg/day to 200 mg/day (122).

X-axis: months from diagnosis. Y-axis: log10 of the percentage of leukemic cells or *BCR::ABL1* (%IS).

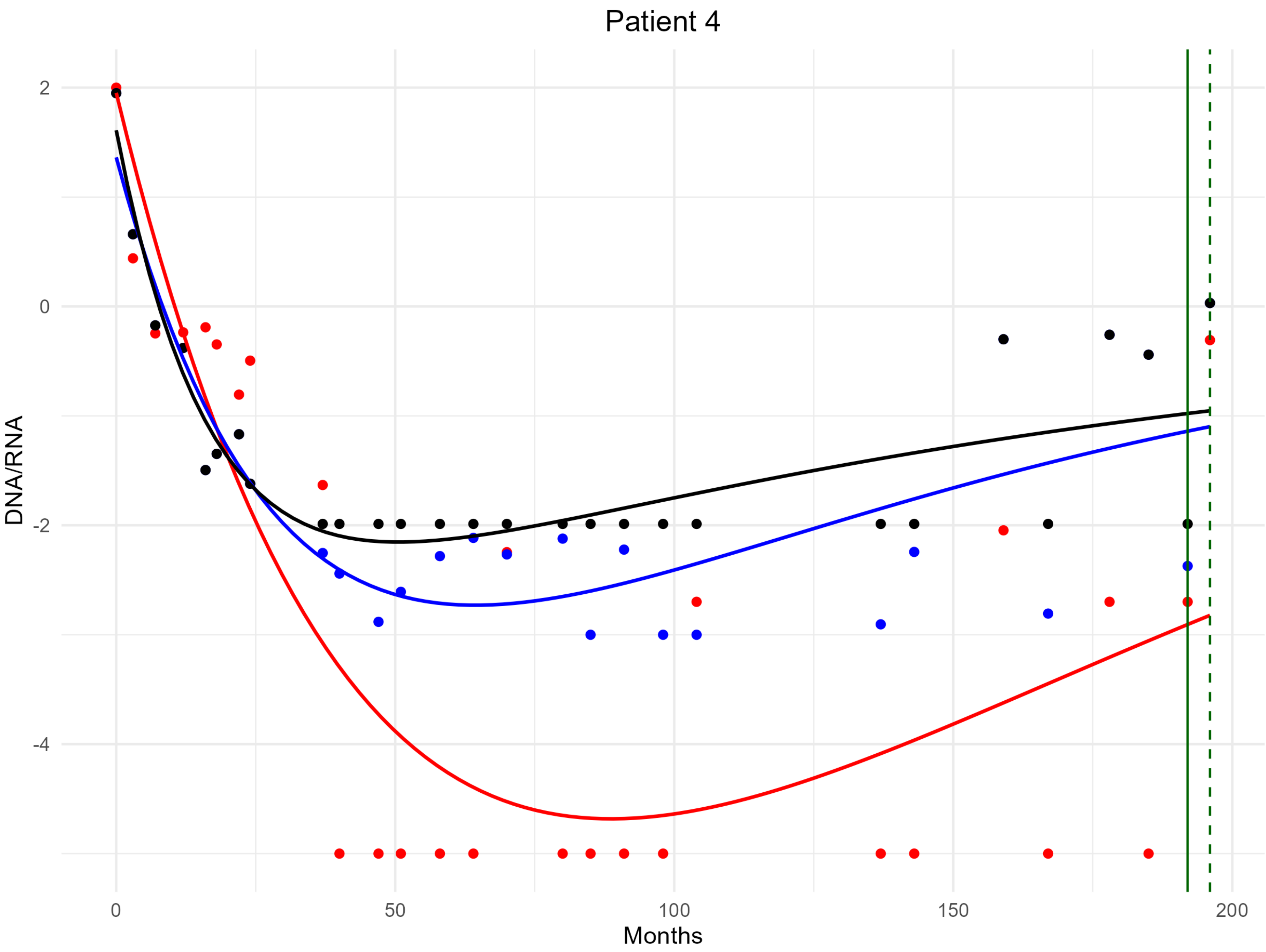

SI4: MRD monitoring of patient 4. Dots represent follow-ups, interpolated by bi-exponential curves; the blue curve is gDNA detected at a sensitivity of 10^{-6}, the red curve is mRNA, the black curve is gDNA detected at a sensitivity of 10^{-4}.

The green solid vertical line indicates the month at which patient interrupted therapy (192); the green dashed vertical line indicates when patient started back TKI assumption (196).

X-axis: months from diagnosis. Y-axis: log10 of the percentage of leukemic cells or *BCR::ABL1* (%IS).

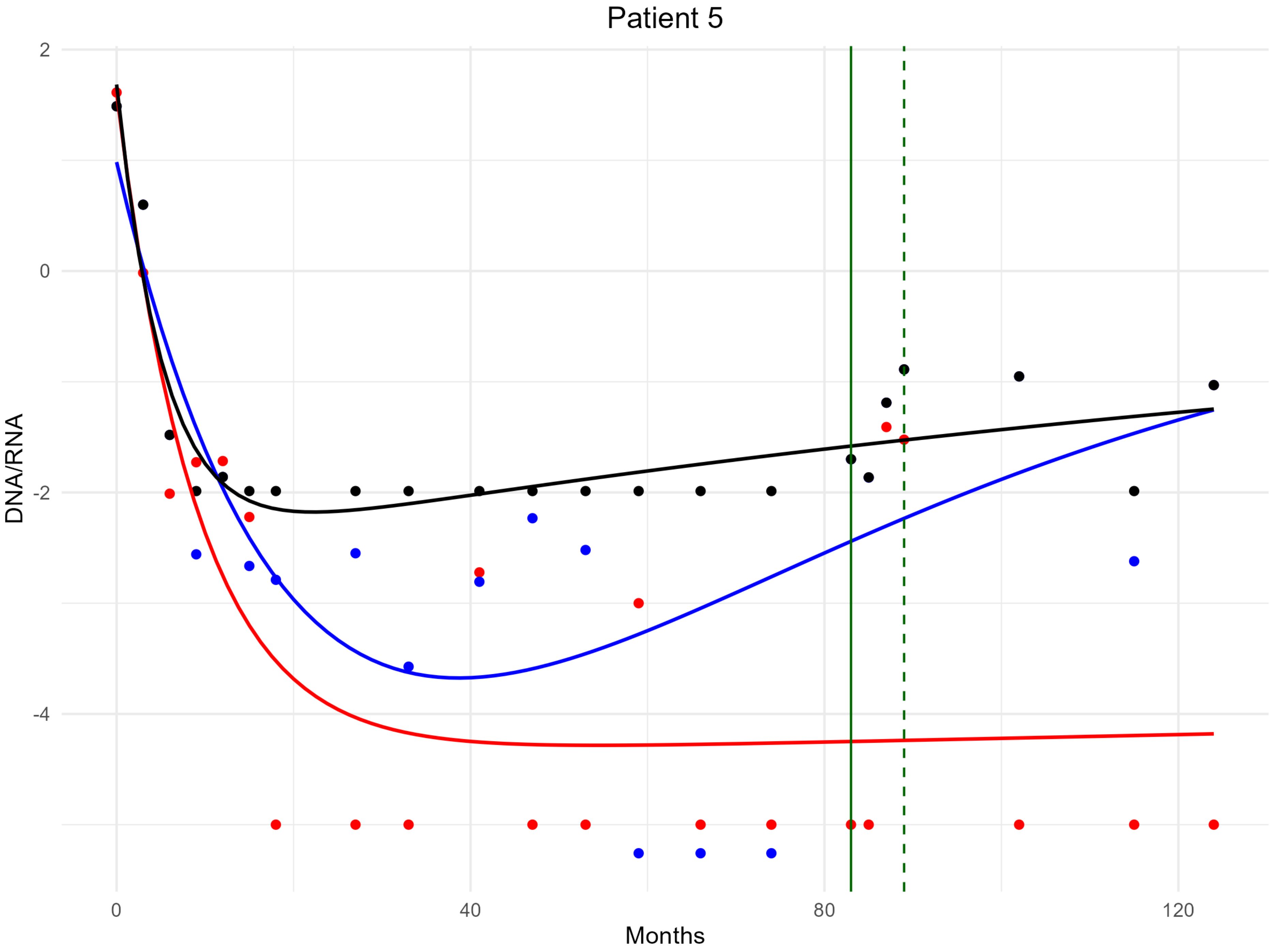

SI4: monitoring of MRD of patient 5.
Dots represent follow-ups, interpolated by bi-exponential curves; the blue curve is gDNA detected at a sensitivity of 10^{-6}, the red curve is mRNA, the black curve is gDNA detected at a sensitivity of 10^{-4}.
The green solid vertical line indicates the month at which patient interrupted therapy (83); the green dashed vertical line indicates when patient started back TKI assumption (89).
X-axis: months from diagnosis. Y-axis: log10 of the percentage of leukemic cells or *BCR::ABL1* (%IS).

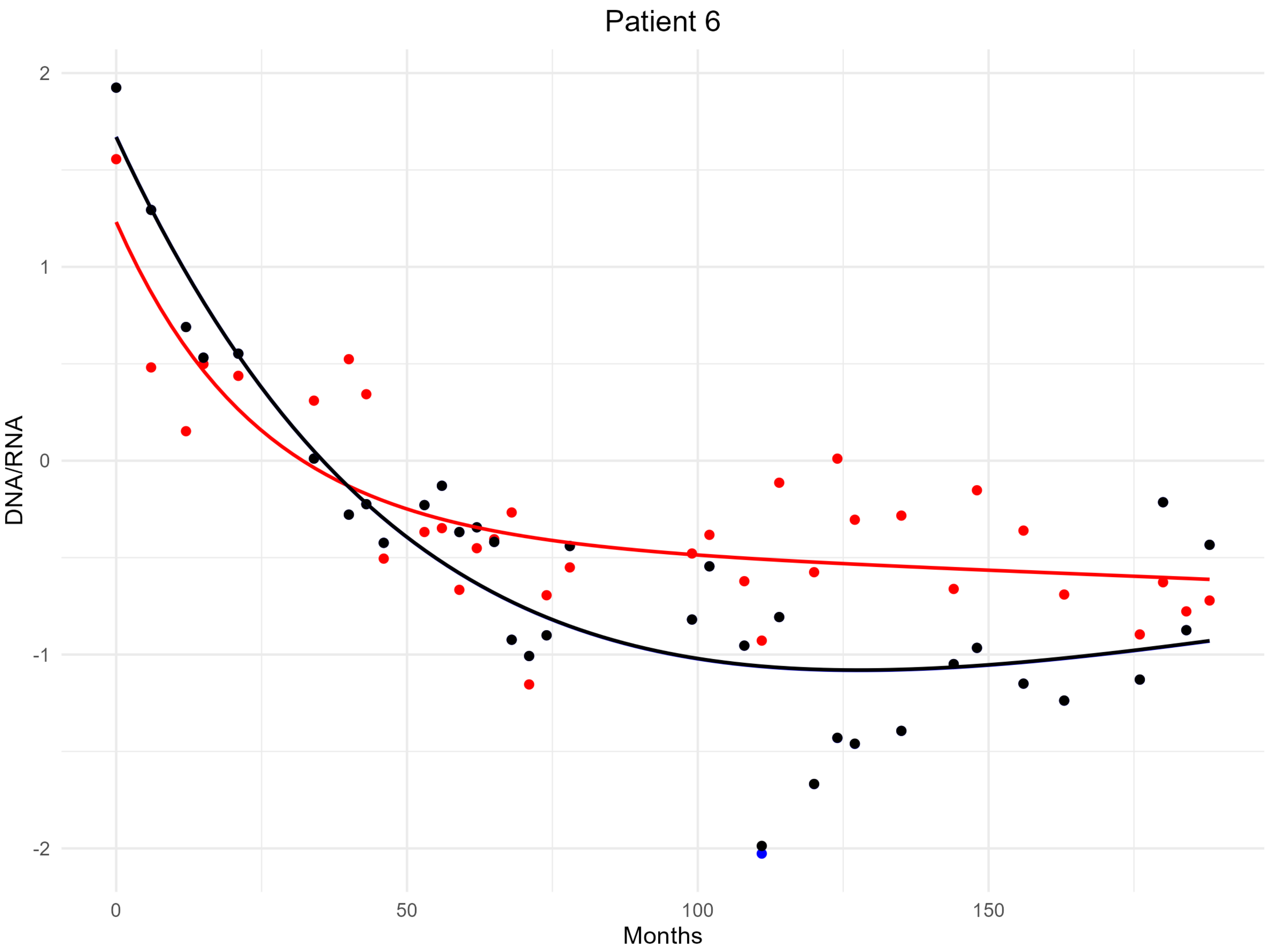

SI4: monitoring of MRD of patient 6.

Dots represent follow-ups, interpolated by bi-exponential curves; the blue curve is gDNA detected at a sensitivity of 10^{-6}, the red curve is mRNA, the black curve is gDNA detected at a sensitivity of 10^{-4}.

Blue and black dots and lines are overlapping.

X-axis: months from diagnosis. Y-axis: log10 of the percentage of leukemic cells or *BCR::ABL1* (%IS).

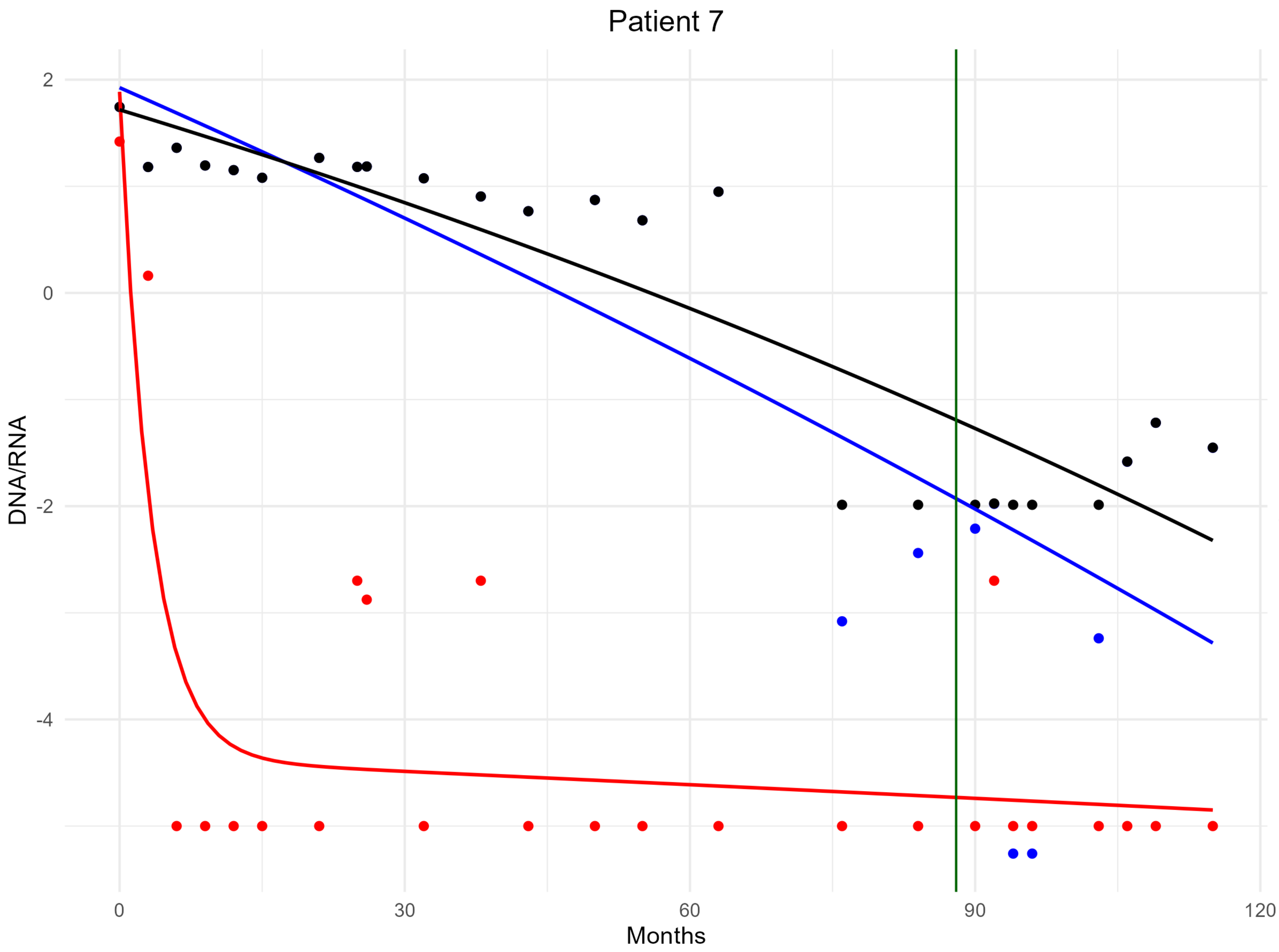

SI4: MRD monitoring of patient 7.
Dots represent follow-ups, interpolated by bi-exponential curves; the blue curve is gDNA detected at a sensitivity of 10^{-6}, the red curve is mRNA, the black curve is gDNA detected at a sensitivity of 10^{-4}.
The green solid vertical line indicates the month at which patient interrupted therapy (88).
X-axis: months from diagnosis. Y-axis: log10 of the percentage of leukemic cells or *BCR::ABL1* (%IS).

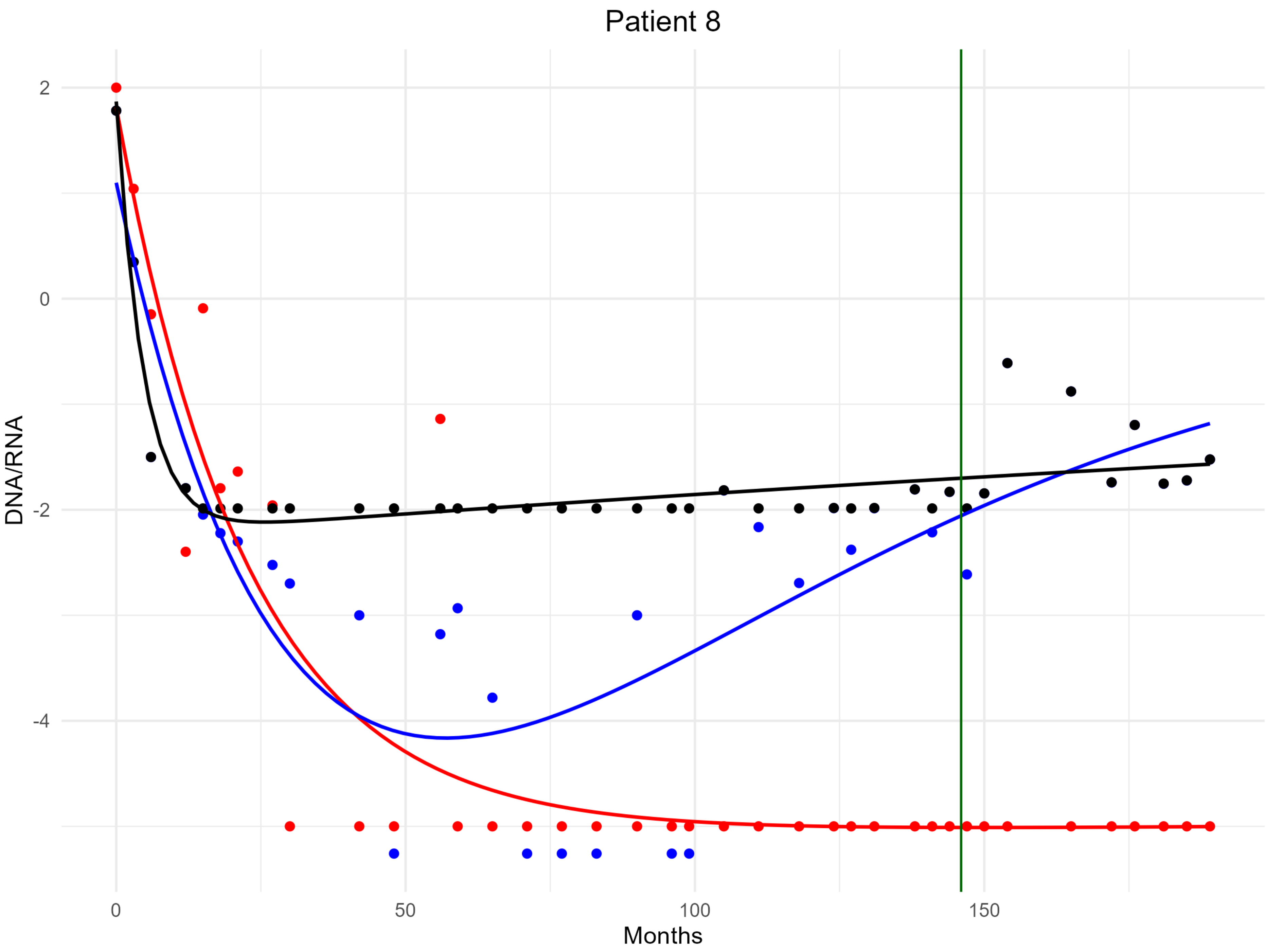

SI4: MRD monitoring of patient 8.

Dots represent follow-ups, interpolated by bi-exponential curves; the blue curve is gDNA detected at a sensitivity of 10^{-6}, the red curve is mRNA, the black curve is gDNA detected at a sensitivity of 10^{-4}.

The green solid vertical line indicates the month at which patient interrupted therapy (146). X-axis: months from diagnosis. Y-axis: log10 of the percentage of leukemic cells or *BCR::ABL1* (%IS).

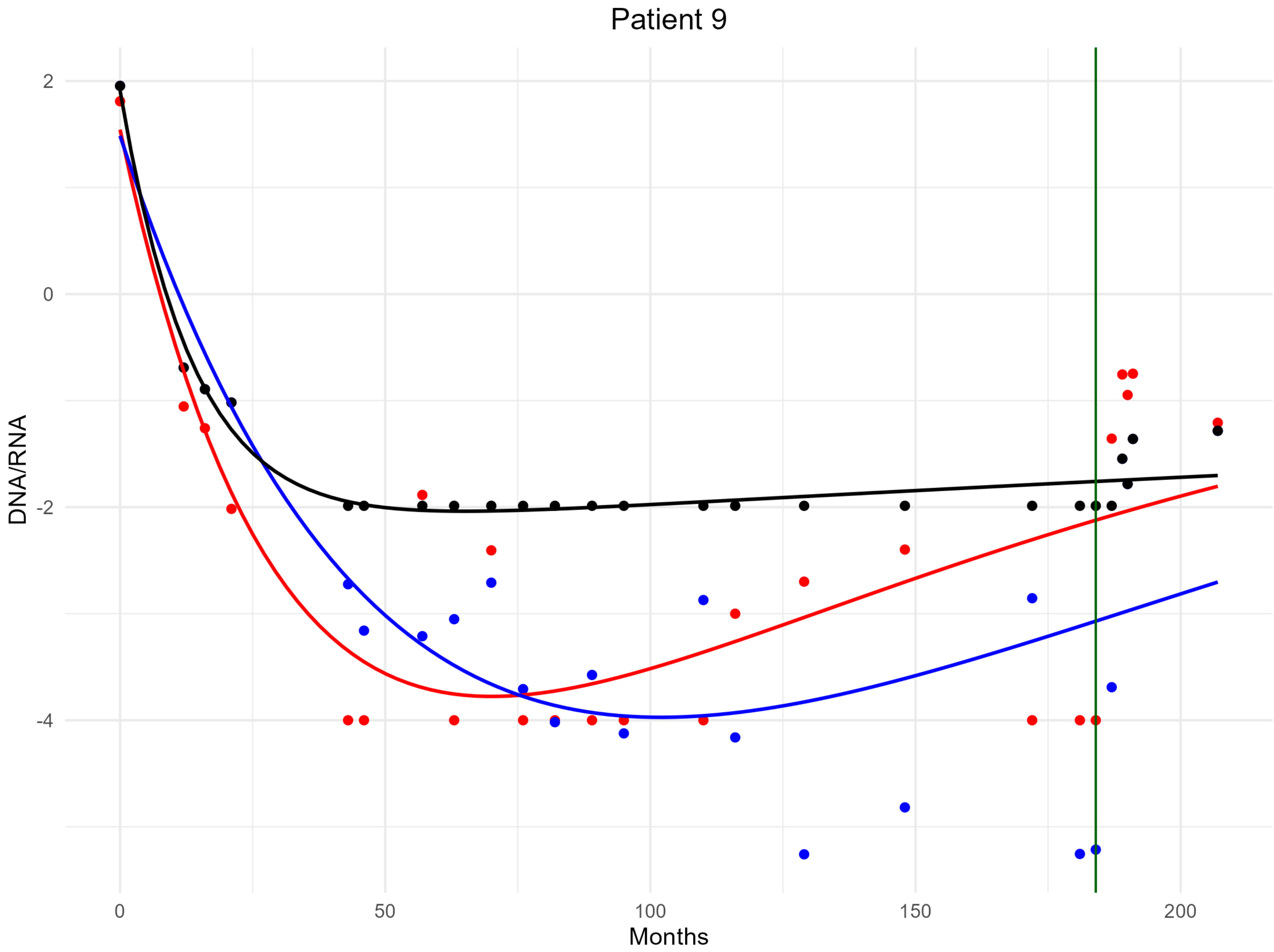

SI4: MRD monitoring of patient 2.

Dots represent follow-ups, interpolated by bi-exponential curves; the blue curve is gDNA detected at a sensitivity of 10^{-6}, the red curve is mRNA, the black curve is gDNA detected at a sensitivity of 10^{-4}.

The green solid vertical line indicates the month at which patient interrupted therapy (184).

X-axis: months from diagnosis. Y-axis: log10 of the percentage of leukemic cells or *BCR::ABL1* (%IS).

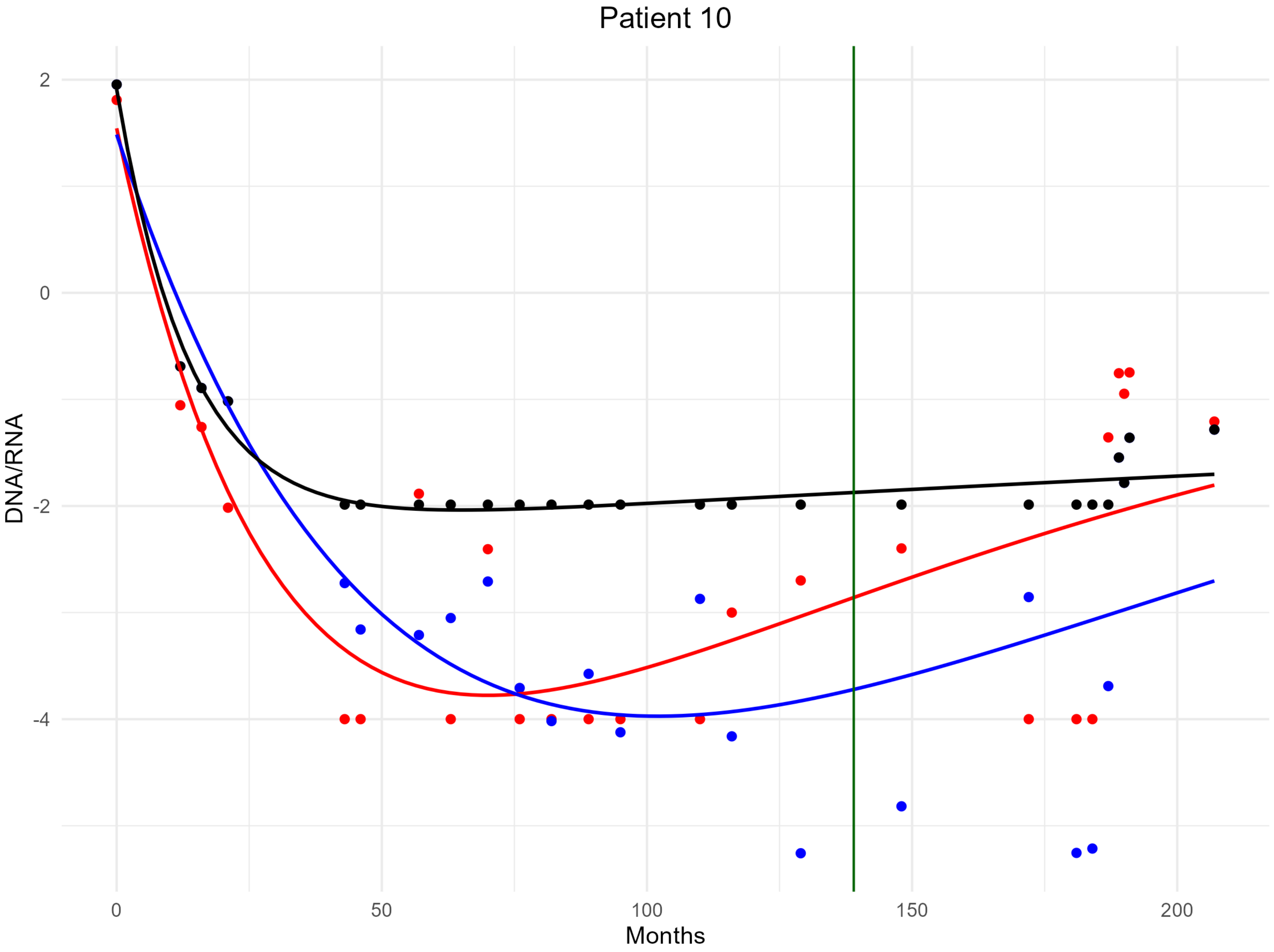

SI4: MRD monitoring of patient 10.
Dots represent follow-ups, interpolated by bi-exponential curves; the blue curve is gDNA detected at a sensitivity of 10^{-6}, the red curve is mRNA, the black curve is gDNA detected at a sensitivity of 10^{-4}.
The green solid vertical line indicates the month at which patient interrupted therapy (139).
X-axis: months from diagnosis. Y-axis: log10 of the percentage of leukemic cells or *BCR::ABL1* (%IS).
